# Effects of hypertonic saline and/or carbocisteine on sputum rheology in bronchiectasis: CLEAR-EME

**DOI:** 10.1183/23120541.00202-2026

**Published:** 2026-09-21

**Authors:** Michael C. McKelvey, Gisli G. Einarsson, Jack Carson, Aram Kadoom, Ryan R. Brown, Christina Campbell, Andrew Jackson, Cliona McDowell, Mike Clarke, Fiona Copeland, Kathryn Ferguson, Rebecca H. McLeese, Rory Convery, Adam T. Hill, Anthony De Soyza, Mitra Shahidi, Martin Kelly, Mary Carroll, Timothy Gatheral, Muhammed Anwar, Jamie Duckers, William Flight, John R. Hurst, A. Alina Ionescu, Michael R. Loebinger, Anita L. Sullivan, James D. Chalmers, Damian G. Downey, Michael M. Tunney, Brenda O'Neill, Daniel F. McAuley, J. Stuart Elborn, Judy M. Bradley, Clifford C. Taggart

**Affiliations:** 1 Wellcome-Wolfson Institute for Experimental Medicine, Queen's University Belfast, Belfast, UK; 2 School of Pharmacy, Queen's University Belfast, Belfast, UK; 3 Northern Ireland Clinical Trials Unit, Belfast Health and Social Care Trust, Belfast, UK; 4 Primary Ciliary Dyskinesia Family Support Group, Ciliopathy Alliance, London, UK; 5 Western Health and Social Care Trust, Londonderry, UK; 6 Wellcome Trust–Wolfson Northern Ireland Clinical Research Facility, Queen's University Belfast, Belfast, UK; 7 Southern Health and Social Care Trust, Craigavon, UK; 8 Department of Respiratory Medicine, Centre for Inflammation Research, University of Edinburgh, Edinburgh, UK; 9 Royal Infirmary of Edinburgh, Edinburgh, UK; 10 Population Health Sciences Institute, Faculty of Medical Sciences, Newcastle University, Newcastle, UK; 11 Buckingham Healthcare NHS Trust, Amersham, UK; 12 University Hospital Southampton NHS Foundation Trust, Southampton, UK; 13 Department of Respiratory Medicine, University Hospitals of Morecambe Bay NHS Foundation Trust, Morecambe Bay, UK; 14 Princess Alexandra Hospital, Harlow, UK; 15 University Hospital Llandough Cardiff and Vale University Health Board, Cardiff, UK; 16 Oxford University Hospitals NHS Foundation Trust, Oxford, UK; 17 UCL Respiratory, University College London, London, UK; 18 Royal Gwent Hospital, Newport, UK; 19 Royal Brompton Hospital, London, UK; 20 National Heart and Lung Institute, Imperial College, London, UK; 21 University Hospitals Birmingham, Birmingham, UK; 22 Scottish Centre for Respiratory Research, Ninewells Hospital and Medical School, University of Dundee, Dundee, UK; 23 Institute of Nursing and Health Research, Ulster University, Belfast, UK

## Abstract

**Background:**

Mucoactives such as hypertonic saline (HTS) and carbocisteine are widely used in the treatment of bronchiectasis, though there is insufficient evidence to support their use. The aim of this mechanistic sub-study, embedded within the CLEAR trial, was to characterise the properties of sputum from patients with bronchiectasis and to assess whether treatment with HTS and/or carbocisteine altered these properties.

**Methods:**

In CLEAR, patients were randomised to receive HTS, carbocisteine, HTS plus carbocisteine or standard care, and sputum samples were collected at randomisation (baseline) and at 2 and 8 weeks post-randomisation. Sputum viscoelasticity was determined by rotational plate rheometry. Biomarkers were quantified by ELISA and microbiome composition was assessed by next-generation sequencing. The primary outcome was differences in sputum viscoelasticity between groups at 2 weeks following the commencement of treatment.

**Results:**

Sputum viscoelastic properties were not reduced by 2 or 8 weeks of treatment with HTS and/or carbocisteine (n=6–15 per group). Sputum biomarker levels and bacterial community composition were similar across groups at 2 or 8 weeks. At baseline, viscoelasticity (crossover point σ_c_) was positively correlated with interleukin-8 (r=0.49, p=0.012) and greater relative bacterial dominance (r=0.35, p=0.041).

**Conclusions:**

These data do not support the use of HTS or carbocisteine to alter sputum viscoelasticity, inflammatory marker levels or bacterial community composition in patients with bronchiectasis.

## Shareable abstract

**Treatment of patients with bronchiectasis with hypertonic saline or carbocisteine does not significantly reduce sputum viscoelastic properties and biomarker levels or alter bacterial community composition** https://bit.ly/4r2X2KA

## Introduction

Bronchiectasis is a chronic respiratory condition characterised by irreversible dilatation and thickening of the airways, which is estimated to affect 50 to 1000 per 100 000 individuals globally, with regional variation in reported prevalence [1–3]. A key feature of bronchiectasis, and a major contributing factor to patient morbidity, is increased mucus production and retention within the airways. In conjunction with airway infection and inflammation, airway remodelling and impaired mucociliary clearance result in a vicious, self-sustaining vortex, which causes progressive lung function decline [4, 5].

The rationale for using mucoactive drugs in the treatment of bronchiectasis is to break this vicious vortex by decreasing the volume of mucus secreted, improving patients’ ability to expectorate sputum or altering mucus viscoelastic properties in such a way that the mucus becomes easier to clear. There is some evidence for the efficacy of certain mucoactives in cystic fibrosis [6, 7] and COPD [8], though there is insufficient evidence to recommend their use in bronchiectasis. Despite this, mucoactives are widely used in the treatment of bronchiectasis [9] and their use is recommended in the European Respiratory Society (ERS) guidelines, though with the acknowledgement that the evidence for their efficacy is limited [10]. Two of the most commonly used mucoactives are nebulised hypertonic saline (HTS) and carbocisteine [9]. In the UK, HTS is used in 6.6% of patients, while carbocisteine or N-acetylcysteine is used in 29.3% of patients [2]. HTS is thought to act primarily as an expectorant, though it also causes acute mucus rehydration by osmosis, drawing water into the airways [11]. Carbocisteine is pleiotropic and its potential modes of action include reducing goblet cell hyperplasia and mucus production, breaking disulfide bonds within the mucus structure and acting as an antioxidant and anti-inflammatory mediator [12, 13].

The CLEAR trial sought to determine the effectiveness of HTS (6% nebulised) and carbocisteine in bronchiectasis over 52 weeks of treatment. The results of the CLEAR trial reported that treatment with HTS or carbocisteine did not significantly reduce the mean number of fully qualifying pulmonary exacerbations over the 52-week period. Secondary outcomes and the incidence of adverse events were also similar across the groups [14].

Sputum can be characterised by its capacity to store energy (elastic modulus G′) or dissipate energy (viscous modulus G″) under stress, and by its ability to resist irreversible deformation (crossover point σ_c_); higher viscoelastic values usually indicate that sputum has a more solid-like and complex structure, which requires the application of large stresses to undergo shear. In the present Efficacy and Mechanism Evaluation sub-study of the CLEAR trial (CLEAR-EME), the primary end-point was to determine whether mucoactive treatment could reduce sputum viscoelasticity. Secondary end-points included determining the effects of mucoactive treatment on sputum biomarker levels and sputum microbiome composition. An exploratory analysis of the relationship between sputum viscoelasticity, biomarker levels and microbiome composition was also performed.

## Methods

Additional details on methods are provided in the supplementary material.

### Main trial design

CLEAR (ISRCTN89040295, NCT04140214) was a 2×2 factorial, randomised, open-label trial conducted across 20 hospitals in the UK. Patients were eligible for inclusion if they were >18 years with a primary diagnosis of bronchiectasis and experienced frequent pulmonary exacerbations (≥2 in the past year, modified to ≥1 in the past year post-Covid-19 pandemic). Patients were randomised (1:1:1:1) to receive: standard care plus twice-daily nebulised HTS (6%); standard care plus carbocisteine (750 mg three times daily until 8 weeks, then 750 mg twice daily); standard care plus HTS and carbocisteine; standard care alone. Full details of the CLEAR trial can be found in the published protocol [15] and main trial manuscript [14].

### EME study design

In the absence of available data from sputum from patients with bronchiectasis, previous findings from a study in which sputum from people with cystic fibrosis was treated with HTS [16] were used to perform a power calculation to determine sample size. A sample size of n=12 per group was calculated to have 90% power to detect a difference in mean±sd elastic modulus of 0.56±0.4. Assuming a 15% drop out and 20% nonadherence rate, the aim was to recruit 72 patients (n=18 per group).

Sequentially enrolled patients recruited to the CLEAR trial were approached to participate in CLEAR-EME. Across eight sites, sputum samples were collected from patients at three timepoints: at the initial visit during which the patient was randomised (baseline); at 2 weeks after the commencement of treatment and at 8 weeks after the commencement of treatment. Samples were spontaneously produced by patients and were collected and immediately stored at −80°C until analysis. Only patients who did not have treatment terminated by 8 weeks were included in the primary and secondary analyses (per protocol population). The funder (National Institute for Health Research (NIHR)) had no role in designing the trial, analysing the data, writing the manuscript, or submitting the manuscript for publication. PARI Pharma provided equipment and consumables in support of the trial but had no role in the design or conduct of the trial or in the analysis or interpretation of the data.

### EME study objectives

The primary objective was to determine differences in viscoelasticity (elastic modulus G′, viscous modulus G″ and crossover point σ_c_) between study groups at 2 weeks after the commencement of treatment.

The secondary objectives were to:

1) determine differences in viscoelasticity (G′, G″ and σ_c_) at 8 weeks after the commencement of treatment;
2) determine differences in sputum biomarkers (interleukin (IL)-6, IL-8 and 8-isoprostane) at 2 and 8 weeks after the commencement of treatment;
3) determine differences in sputum bacterial community composition at 2 and 8 weeks after the commencement of treatment.

Additional *post hoc* analysis was also conducted focussing on two main groupwise comparisons: patients who received HTS compared to patients who did not receive HTS, and patients who received carbocisteine compared to patients who did not receive carbocisteine.

### Sputum sample processing

Whole sputum was thawed in a sterile petri dish, and mucus was isolated from any liquid-phase contaminants (*e.g.* saliva) by manipulation with forceps. Isolated mucus was gently homogenised by nondestructive vortexing [17] and then divided into three aliquots. The first aliquot was used for rheometry, the second was processed in Dulbecco's PBS for ELISA and the third was retained for bacterial analysis. Where insufficient quantities of sputum were available for all three analyses, rheometry was prioritised as this was the primary objective of the study.

### Rheometry

Sputum rheometry was performed on the Rheomuco rotational plate rheometer (Rheonova, France) as per the manufacturer's recommendations.

### Enzyme-linked immunosorbent assays

DPBS-processed sputum samples were run on commercially available ELISA kits for IL-6 (88-7066; Invitrogen, MA, USA), IL-8 (DY208; R&D Systems, MN, USA) and 8-isoprostane (CAY516351; Cayman Chemical, MI, USA), as per the manufacturer's instructions.

### Sputum pre-lysis and DNA extraction for bacterial analysis

Sputum preparation for bacterial analysis was performed as has previously been reported [18]. Briefly, an aliquot of sputum was pre-lysed using sequential physical and chemical disruption. Nucleic acids were extracted and purified using the MagNA Pure 96 system (Roche, UK).

### 16S rRNA sequencing

Illumina MiSeq (Illumina, CA, USA) sequencing targeting the 16S rRNA marker gene V4 region was performed using modified universal primers based on the Earth Microbiome protocol [19]. Diversity analyses including measures of α-diversity (Shannon–Wiener diversity, Pielou's evenness, richness and relative dominance) were determined.

### Statistical analysis

Analyses were conducted on the population of patients recruited to CLEAR-EME who received the study drugs up to at least week 8 (time of final sputum sample collection) unless otherwise stated. For the primary outcome measure, comparisons were made between groups using Analysis of Covariance (ANCOVA) to detect differences in means adjusted for baseline values (with Tukey's Honestly Significant Difference *post hoc* test when significant differences were identified). For secondary outcomes, inferential statistics were not performed, and data were reported as mean±95% confidence intervals (95% CI). Correlations (Spearman r) between rheometry outputs, biomarkers and bacterial community indices were performed for all patients for whom data were available. Data were analysed using Stata version 15.1 (StataCorp, TX, USA), Prism version 10.6.0 (GraphPad, MA, USA) and R version 4.1.2 (https://www.r-project.org/). p-values <0.05 were considered statistically significant.

## Results

**FIGURE 1:**
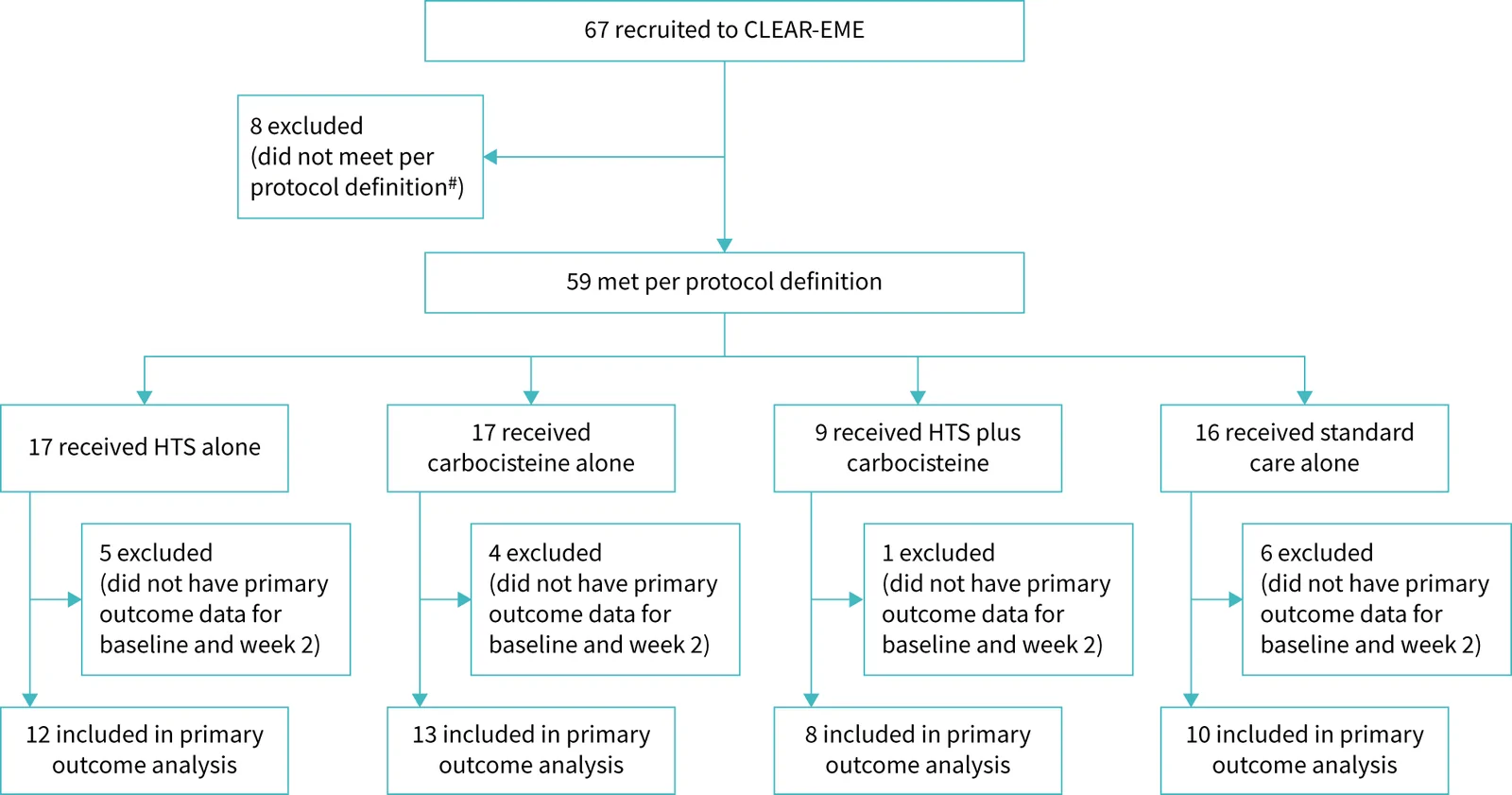
Study flow diagram for CLEAR-EME. Participants in CLEAR were randomised 1:1:1:1 to one of four treatment arms (hypertonic saline (HTS) alone, carbocisteine alone, HTS plus carbocisteine or standard care alone). Of the 67 recruited to CLEAR-EME, eight were excluded who did not meet the per protocol definition. A further 16 were excluded due to the absence of primary outcome data (rheometry at baseline and 2 weeks). ^#^: patients who did not have the study drug terminated within the first 8 weeks.

Between 25 January 2022 and 28 September 2023, 67 patients were recruited to CLEAR-EME, across eight sites in the UK, which was below the *a priori* target sample size of 72 patients. As presented in the flow diagram in figure 1, of the 67 recruited patients, 59 met the per protocol inclusion criteria (study drug received up to week 8), and 43 patients had primary outcome data (*i.e.* sputum rheometry at baseline and 2 weeks).

Baseline characteristics for the per protocol population with available primary outcome data are detailed in table 1. Baseline characteristics for the whole CLEAR-EME cohort can be found in supplementary table S1.

**TABLE 1:** Baseline characteristics at trial entry (EME per protocol population with primary outcome data)

Baseline characteristics	Treatment group			
	HTS	Carbocisteine	HTS plus carbocisteine	Standard care
**Patients, n**	12	13	8	10
**Sex**				
Male	9 (75.0%)	4 (30.8%)	5 (62.5%)	2 (20.0%)
Female	3 (25.0%)	9 (69.2%)	3 (37.5%)	8 (80.0%)
**Age years**	60.7±15.3	68.8±13.6	66.5±13.8	65.7±12.3
**Ethnicity**				
White	11 (91.7%)	13 (100.0%)	8 (100.0%)	9 (90.0%)
Asian/Asian British	1 (8.3%)	0 (0.0%)	0 (0.0%)	0 (0.0%)
Chinese	0 (0.0%)	0 (0.0%)	0 (0.0%)	1 (10.0%)
**Cigarette use**				
Never smoked	4 (33.3%)	8 (61.5%)	5 (62.5%)	5 (50.0%)
Ex-Smoker				
Cigarettes	7 (58.3%)	5 (38.5%)	3 (37.5%)	5 (50.0%)
Pipe	1 (8.3%)	0 (0.0%)	0 (0.0%)	0 (0.0%)
**E-cigarette use – never vaped**	12 (100.0%)	13 (100.0%)	8 (100.0%)	10 (100.0%)
**Weight kg**	84.5±13.1	74.2±21.8	81.7±23.3	76.6±17.2
**BSI**	5.2±3.2	8.3±3.5 n=12	6.4±4.0	7.0±2.9
**FACED**	1.1±1.2	2.5±1.9 n=12	1.5±1.3	1.6±1.1
**HRQoL-B (Respiratory Domain)**	64.2±14.5	52.4±20.4	64.8±14.4	61.9±12.2

Data are presented as mean±sd for continuous variables and n (%) for all categorical variables. If n is different from the total n per group (due to missing data) it is noted in the relevant cell. HTS: hypertonic saline; BSI: Bronchiectasis Severity Index; FACED: FEV_1_, age, chronic colonisation, extension, dyspnoea; HRQoL-B: Health-Related Quality of Life–Bronchiectasis score.

There was variation in the sex representation between groups with a higher proportion of males in the HTS and HTS plus carbocisteine groups and a higher proportion of females in the carbocisteine and standard care groups. The HTS group was the youngest, while the carbocisteine group was the oldest. The carbocisteine group had the highest Bronchiectasis Severity Index and FACED scores.

### Primary outcome measure

**FIGURE 2:**
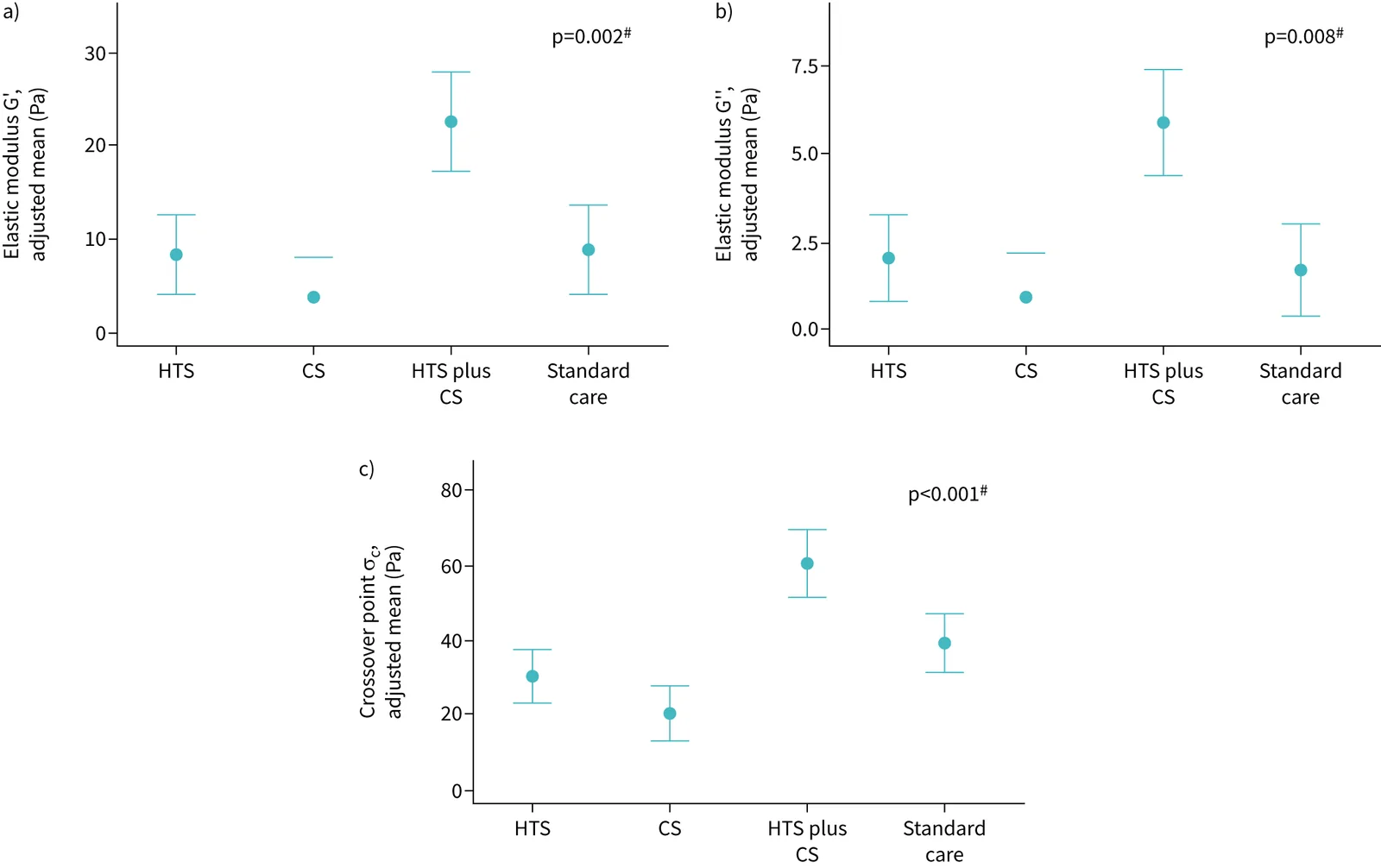
Sputum viscoelasticity at 2 weeks after commencement of treatment. Sputum viscoelastic properties were determined using a rotational plate rheometer. Comparisons of a) elastic modulus G′, b) viscous modulus G″ and c) crossover point σ_c_ were made between values at 2 weeks adjusted for baseline. Data were analysed by ANCOVA and are presented as mean±se. p-values are from ANCOVA and p<0.05 denotes statistical significance. ^#^: Tukey's Honestly Significant Difference pairwise comparisons did not identify any significant differences. HTS: hypertonic saline; CS: carbocisteine.

After adjustment for baseline sputum viscoelasticity, there was no evidence of reduced viscoelasticity following 2 weeks of treatment with HTS and/or carbocisteine compared with standard care (figure 2). ANCOVA identified differences in elastic modulus G′ (p=0.002), viscous modulus G″ (p=0.008) and crossover point σ_c_ (p<0.001) between the four groups. This appears to have been driven by elevated viscoelasticity in the HTS plus carbocisteine group, with no reduction in viscoelasticity observed in the other mucoactive treatment arms. *Post hoc* pairwise comparisons did not identify significant differences. Full data are presented in supplementary table S2.

### Secondary outcome measures

As samples were prioritised for primary outcome analysis, secondary outcome data were unavailable for all patients. Due to the limited sample size, inferential testing was not performed for secondary outcomes, though data appear to be similar across the groups regarding sputum viscoelasticity at 8 weeks, inflammatory and oxidative stress biomarkers at 2 and 8 weeks, and bacterial community composition at 2 and 8 weeks. These data are presented in supplementary tables S3–S7.

Analysis of the sputum microbiome revealed numerical variation in diversity indices; however, these differences were not statistically significant at baseline across the groups (supplementary figure 1A). These differences were stable across study time points (supplementary figure 1B), regardless of treatment group, indicating that baseline bacterial community composition was the dominant predictor of values at later time points. Furthermore, the bacterial taxa present, and their relative abundance also appeared to be stable, regardless of treatment group (supplementary figure 2).

### *Post hoc* groupwise analysis

**FIGURE 3:**
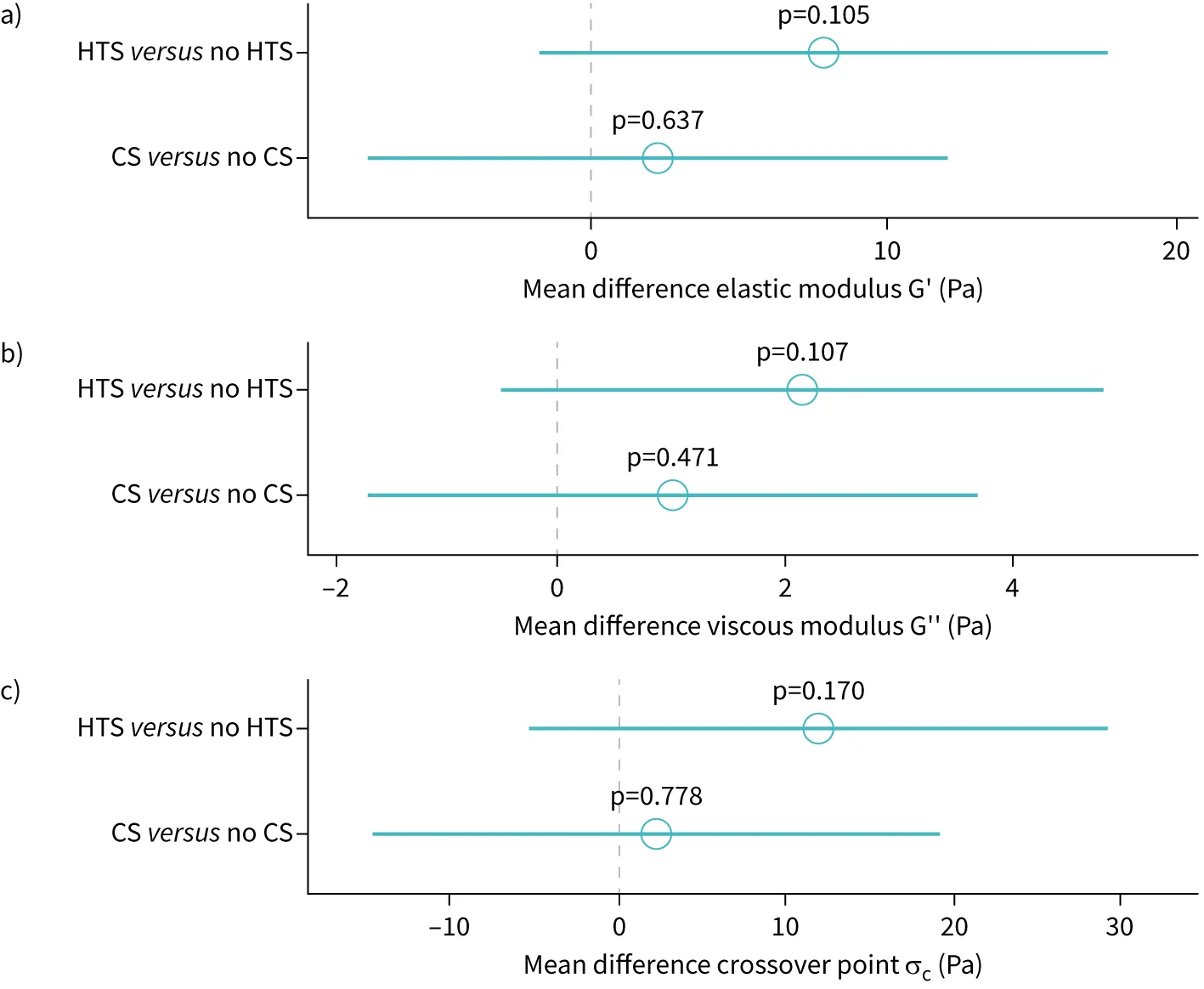
Groupwise comparison of the effects of hypertonic saline (HTS) and carbocisteine (CS) on sputum viscoelasticity at 2 weeks after the commencement of treatment. Forest plots showing the mean differences and 95% confidence intervals for rheological parameters between treatment groups. The dashed vertical line represents zero mean difference (no effect). a) Mean difference in elastic modulus G′; comparison of HTS *versus* no HTS (p=0.105) and CS *versus* no CS (p=0.637). b) Mean difference in viscous modulus G″; comparison of HTS *versus* no HTS (p=0.107) and CS *versus* no CS (p=0.471). c) Mean difference in crossover point σ_c_; comparison of HTS *versus* no HTS (p=0.170) and CS *versus* no CS (p=0.778). In all measured parameters, the 95% CIs cross the zero-difference line, indicating no statistically significant difference (at p>0.05) was observed for the HTS or carbocisteine treatments when compared to their respective controls.

To align the results of CLEAR-EME with the main CLEAR trial, *post hoc* analyses were performed involving two separate comparisons: participants receiving HTS *versus* those who were not receiving HTS and participants receiving carbocisteine *versus* those who were not receiving carbocisteine. No interactions were anticipated between HTS and carbocisteine, and this was formally tested as part of the main trial analysis; no evidence of interaction was found [14]. In these group comparisons, there was no statistically significant difference in sputum viscoelasticity at 2 weeks with either HTS or carbocisteine (figure 3 and supplementary table S8). Numerically higher mean viscoelasticity was observed in the group receiving HTS compared to no HTS, and the group receiving carbocisteine compared to no carbocisteine.

**FIGURE 4:**
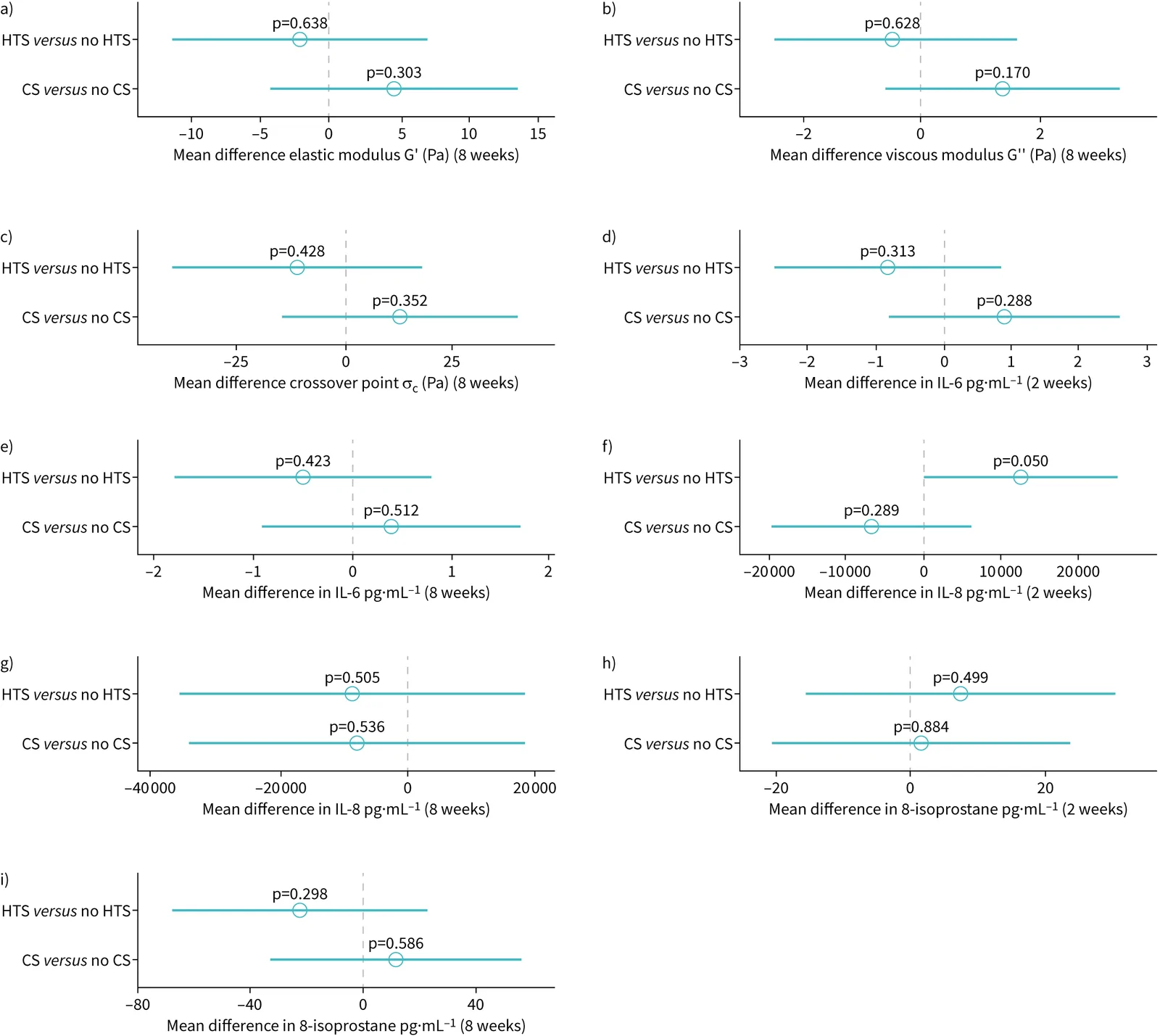
Groupwise comparison of the effects of hypertonic saline (HTS) and carbocisteine (CS) on sputum viscoelasticity at 8 weeks and biomarkers at 2 and 8 weeks after the commencement of treatment. Forest plots showing the mean differences and 95% confidence intervals for parameters between treatment groups. The analysis focuses on two primary comparisons: HTS *versus* no HTS and CS *versus* no CS. The dashed vertical line represents zero mean difference (no effect). Plots represent mean difference in a) elastic modulus G′, b) viscous modulus G″ and c) crossover point σ_c_ at 8 weeks, and d and e) interleukin (IL)-6, f and g) IL-8 and h and i) 8-isoprostane at 2 and 8 weeks. In all measured parameters, the 95% CIs cross the zero-difference line, indicating no statistically significant difference (p>0.05).

Further analyses (rheometry at 8 weeks and biomarkers at 2 and 8 weeks) also showed no significant differences with either HTS or carbocisteine (figure 4). Values for groupwise comparisons, including bacterial diversity analyses, can be found in supplementary table S9.

### Baseline sputum correlations

**FIGURE 5:**
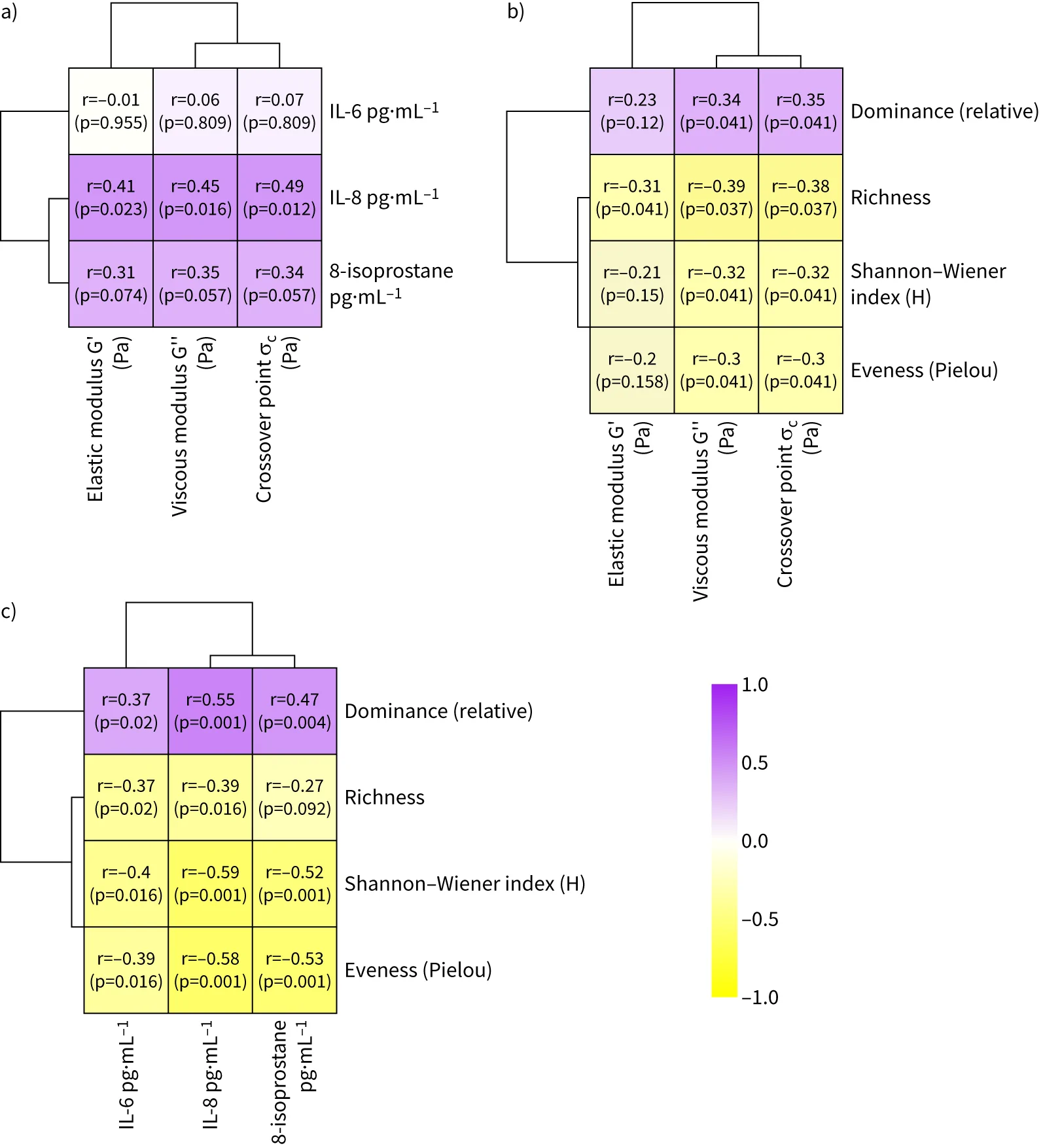
Baseline correlations in sputum from patients with bronchiectasis. Sputum viscoelastic properties (elastic modulus G′, viscous modulus G″ and crossover point σ_c_) were correlated with a) markers of inflammation (interleukin (IL)-6 and IL-8) and oxidative stress (8-isoprostane), and b) α-diversity indices of bacterial community composition (Shannon–Wiener diversity, Pielou's evenness, richness and relative dominance). c) Biomarkers were correlated with indices of bacterial community composition. Each cell contains the Spearman correlation coefficient (r) and the corresponding p-value. The colour intensity represents the magnitude and direction of the correlation, with purple indicating positive correlations and yellow indicating negative correlations. Paired rheometry and α-diversity indices of bacterial community composition were available for n=51; biomarker and/or rheometry and α-diversity indices of bacterial community composition data were available for n=41 patients. Correlations were considered statistically significant if p<0.05.

To characterise the relationship between sputum rheometry, biomarker levels and bacterial community composition, correlations were performed on data from baseline sputum samples. Correlations were performed for all available samples (including those from patients that did not meet the per protocol definition and those excluded from the primary and secondary analyses due to an absence of week 2 and week 8 data) and are presented in figure 5. IL-8 was positively correlated with elastic modulus G′ (r=0.41, p=0.023), viscous modulus (r=0.45, p=0.016) and crossover point σ_c_ (r=0.49, p=0.012), while crossover point σ_c_ was also positively correlated with relative bacterial dominance (r=0.35, p=0.041) and negatively correlated with Shannon–Wiener diversity (r=0.32, p=0.041), Pielou's evenness (r=0.30, p=0.041) and species richness (r=0.38, p=0.037). Levels of biomarkers were positively correlated with relative dominance and negatively correlated with diversity, evenness and richness. Overall, pairwise correlations for samples collected at 2 weeks and 8 weeks were similar to those at baseline and are presented in supplementary figure S3.

## Discussion

The present mechanistic sub-study of the CLEAR trial is the first study to examine whether HTS or carbocisteine are effective in altering the rheological properties of sputum in patients with bronchiectasis. We report that there was no benefit to the use of nebulised HTS or oral carbocisteine in patients with bronchiectasis in terms of reducing sputum viscoelasticity. Indeed, treatment with HTS and carbocisteine appeared to increase viscoelasticity at 2 weeks, though *post hoc* pairwise comparisons did not find these differences to be statistically significant, presumably due to the heterogeneity of the data. Moreover, there was no apparent difference in the levels of sputum markers of inflammation or oxidative stress, or differences in the sputum bacterial community composition between groups. However, these findings should be interpreted with caution as we acknowledge that due to a shortfall in the number of patients recruited and the necessary exclusion of patients (due to treatment termination or lack of sample), the power of the study to determine the prespecified outcomes is reduced.

A key pathological feature of bronchiectasis is the excessive production of mucus with enhanced viscoelasticity. Similar to reports in a variety of other chronic respiratory diseases [20, 21], sputum viscoelasticity in the CLEAR-EME cohort was dominated by the elastic component (elastic modulus G′), which was consistently higher than the viscous component (viscous modulus G″). As expected, the crossover point σ_c_, at which sputum begins to flow under stress, was up to 20-fold higher than that of healthy sputum and comparable to that of other bronchiectasis cohorts [20, 22]. In the context of bronchiectasis, where patients often struggle to produce high cough velocities, the elevated stresses required to shear such ductile mucus may not be attainable [23]. The failure to clear mucus by conventional biomechanics (mucociliary clearance, cough, *etc.*) leads to its accumulation in the airways; this causes obstruction of the airways and provides a fertile environment for pathogen colonisation, promoting inflammation, and contributing to exacerbations and lung function decline [5].

In terms of mucus management in bronchiectasis, guidelines recommend that airway clearance techniques be employed to help ameliorate symptoms [9, 24, 25]. Mucoactives (expectorants, mucoregulators, mucolytics and mucokinetics) also form a major part of treatment. The recently published CLEAR trial sought, in a randomised controlled trial setting, to assess whether the addition of nebulised HTS or oral carbocisteine to standard care (which included airway clearance techniques) provided any clinical benefit to patients with bronchiectasis. The trial reported no between-group differences in terms of number of pulmonary exacerbations over 52 weeks, or numerous secondary outcomes including health-related quality of life and lung function [14]. Therefore, although HTS and carbocisteine can be considered safe, in the absence of apparent biological (as reported here) and clinical [14] efficacy, the substantial cost and treatment burden associated with these treatments should encourage clinicians to reconsider their routine prescription for the broad spectrum of patients with bronchiectasis [26].

Previous studies have variously characterised the sputum viscoelastic properties [17, 27], sputum microbiome [18, 28, 29] and sputum biomarker levels [30, 31] in patients with bronchiectasis. However, CLEAR-EME provided an opportunity to correlate these measures in a cohort of patients with active but stable bronchiectasis. In both the linear/rest phase (elastic modulus G′, viscous modulus G″) and nonlinear/flow phase (crossover point σ_c_), sputum rheometry was correlated with indices of inflammation and bacterial community dysbiosis. These findings are consistent with the concept that sputum with a more solid-like composition and a more cohesive, cross-linked internal structure is associated with markers of pathobiology in bronchiectasis. Although we were unable to perform rheometry on samples immediately after collection, it has been shown that timely storage of fresh samples at −80°C preserves rheological properties [17].

A strength of the present study is that we considered rheology alongside biomarkers and microbiome analysis. Many drugs that are referred to as mucoactives are pleiotropic, so it is possible that their main mechanism of disease alteration is not by acting on the mucus itself [13, 32]. Three biomarkers were selected for analysis. IL-6 (a marker of inflammation) and 8-isoprostane (a marker of oxidative stress) have previously been shown to be reduced in patients with COPD receiving carbocisteine treatment [33], while IL-8 (a marker of neutrophilic inflammation) is reduced in sputum from patients with cystic fibrosis receiving HTS [34]. The levels of all three are known to be elevated in the lungs of patients with bronchiectasis [35, 36]. Although the limited sample size precludes any definitive inferences, that the results appear similar across groups suggests that HTS and carbocisteine do not meaningfully alter inflammation, oxidative stress or bacterial community composition. This sample size limitation was especially evident in analysis of secondary outcomes, where there was often insufficient sample volume remaining after rheological analysis to complete biomarker quantification and microbiome analysis. Thus, caution must be exhibited when interpreting these data and a larger study would be required to confirm that mucoactive therapy has no effect on these secondary outcomes.

Patients recruited to CLEAR were required to produce daily sputum, though there were no inclusion criteria relating to sputum quantity or difficulty in clearance [15], so it is possible that the results would differ if subgroups were selected according to these characteristics. We also cannot exclude the possibility that HTS and/or carbocisteine increase the volume of sputum cleared; these data were not captured. All participants in CLEAR either already utilised airway clearance techniques or were trained to do so as part of standard care. It is possible that airway clearance competency may have an impact on the variables measured in this study, though this is not known since competency was not assessed in the main trial.

This study focussed on sputum rheology and does not address other sputum properties that may be impacted, such as percentage solids, DNA and changes in mucin proteins. These properties are known to contribute to the overall rheological performance of sputum [37, 38], and further studies will be required to address whether these individual properties are altered following mucoactive treatment. Although we have shown that neither HTS nor carbocisteine meaningfully alter the rheological properties of expectorated sputum after 2 or 8 weeks of treatment, it is possible that changes may have been seen over a longer treatment period. However, the combination of the absence of clinical benefit over 52 weeks and no clear biological effect up to 8 weeks gives us confidence that these treatments are not effective mucoactives in this population. Another consideration is that the CLEAR trial was a UK-based trial, potentially limiting the generalisability of our findings to the wider bronchiectasis population in terms of, for example, geographical location, which is known to affect which pathogens predominate [29]. Our baseline analysis of the sputum microbiome would indicate that the CLEAR-EME population was typical of a stable UK cohort, with a predominance of *Haemophilus influenzae* [2, 29]. Previous studies have reported the stability of the sputum microbiome in bronchiectasis over time [39–41], and such stability was a feature of the CLEAR-EME cohort. Since patients who were actively exacerbating were not recruited to CLEAR, we cannot comment on whether a different treatment effect may have been seen in exacerbating patients, or those who had, for example, a *Pseudomonas aeruginosa-*dominated microbiome.

It is important to note that the findings of CLEAR and CLEAR-EME do not preclude efficacy of other mucoactive treatments, such as erdosteine or N-acetylcysteine, which should be carefully assessed in similar large, randomised trials. Further work is required to understand the structural composition of airway mucus in patients with bronchiectasis so that targeted mucoactive therapy can be selected. For example, patients with highly cross-linked mucus may benefit more from treatment with mucolytics [12]. Aside from selecting mucoactives by mode of action, it is important to validate that these treatments are actually effective in terms of *in vivo* penetrance into the airways and the mucus plugs themselves; most studies showing reduced viscoelasticity with mucoactives are performed *ex vivo*, where mucoactives are actively mixed with sputum [16, 42, 43], which is not representative of the physiological scenario.

In summary, our data indicate that neither HTS nor carbocisteine effectively reduce sputum viscoelasticity in patients with bronchiectasis. They also do not appear to alter inflammation, oxidative stress or sputum microbiome composition. These findings build on the results of the CLEAR trial and provide a possible mechanistic explanation for the lack of clinical efficacy of these treatments.

## Supplementary material

**Please note:** supplementary material is not edited by the Editorial Office, and is uploaded as it has been supplied by the author. Supplementary material 00202-2026.SUPPLEMENT
