# “Effects of hypertonic saline and/or carbocisteine on sputum rheology in bronchiectasis: CLEAR-EME” Michael C. McKelvey, Gisli G. Einarsson, Jack Carson, Aram Kadoom, Ryan R. Brown, Christina Campbell, Andrew Jackson, Cliona McDowell, Mike Clarke, Fiona Copeland, Kathryn Ferguson, Rebecca H. McLeese, Rory Convery, Adam T. Hill, Anthony De Soyza, Mitra Shahidi, Martin Kelly, Mary Carroll, Timothy Gatheral, Muhammed Anwar, Jamie Duckers, William Flight, John R. Hurst, A. Alina Ionescu, Michael R. Loebinger, Anita L. Sullivan, James D. Chalmers, Damian G. Downey, Michael M. Tunney, Brenda O'Neill, Daniel F. McAuley, J. Stuart Elborn, Judy M. Bradley and Clifford C. Taggart on behalf of the CLEAR study group. *ERJ Open Res* 2026; 12: 00202-2026.

**DOI:** 10.1183/23120541.50202-2026

**Published:** 2026-10-12

This article was originally published with an error in figure 2b in which the vertical axis label incorrectly read “Elastic modulus” rather than “Viscous modulus”. The corrected figure is shown below. This has also been corrected in the article itself.

**FIGURE 2:**
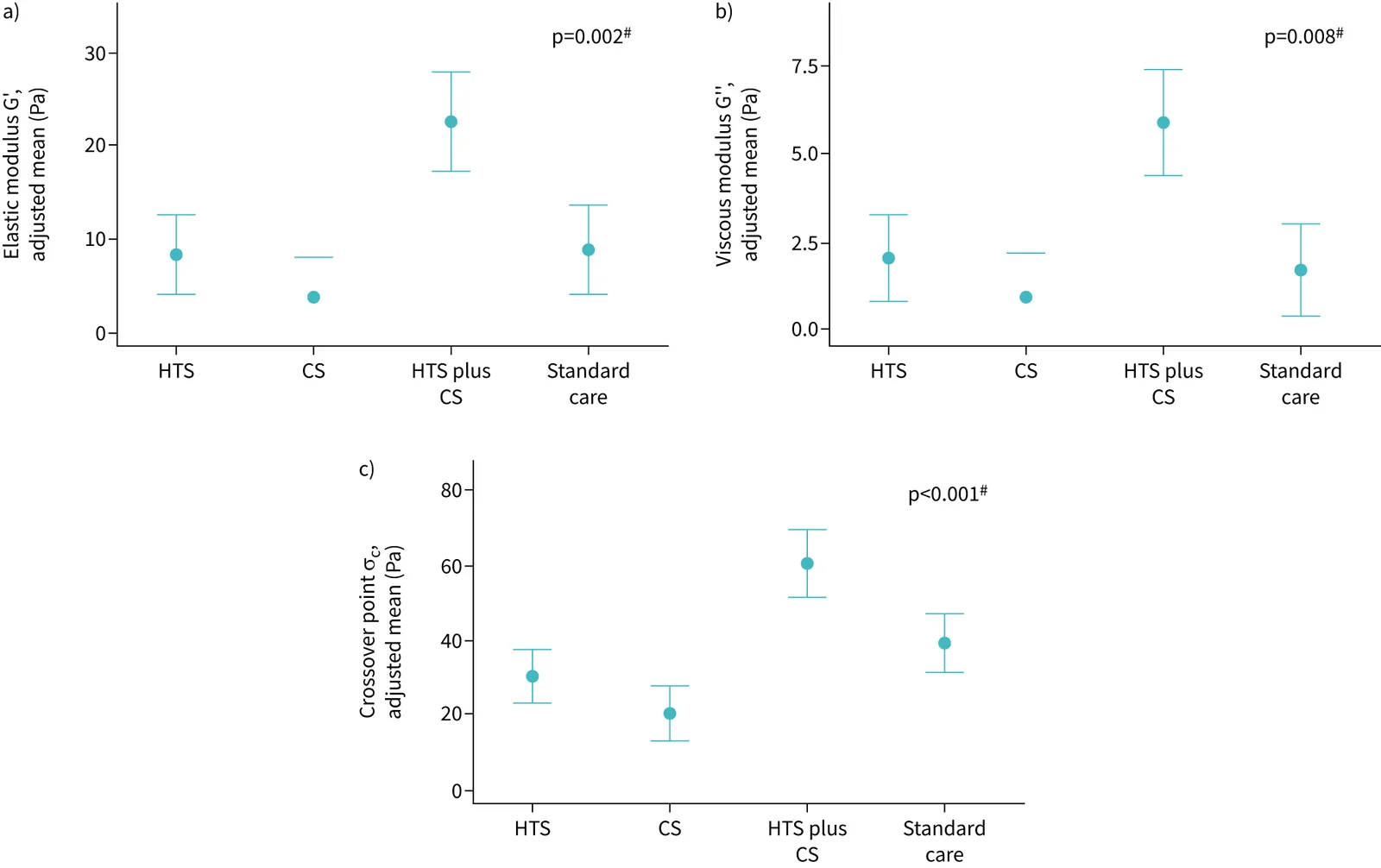
Sputum viscoelasticity at 2 weeks after commencement of treatment. Sputum viscoelastic properties were determined using a rotational plate rheometer. Comparisons of a) elastic modulus G′, b) viscous modulus G″ and c) crossover point σ_c_ were made between values at 2 weeks adjusted for baseline. Data were analysed by ANCOVA and are presented as mean±se. p-values are from ANCOVA and p<0.05 denotes statistical significance. ^#^: Tukey's Honestly Significant Difference pairwise comparisons did not identify any significant differences. HTS: hypertonic saline; CS: carbocisteine.

