# Supplementary material for: Effects of hypertonic saline and/or carbocisteine on sputum rheology in bronchiectasis: CLEAR-EME
Source: ERJ Open Res. 2026 Sep 21;12(5):00202-2026. doi: 10.1183/23120541.00202-2026 (PMC13591285; doi:10.1183/23120541.00202-2026)
Supplement: Supplementary file 1 [file 00202-2026.SUPPLEMENT.pdf]

Effects of hypertonic saline and/or carbocisteine on sputum rheology in bronchiectasis (CLEAR-EME)

Michael C McKelvey, Gisli G Einarsson, Jack Carson, Aram Kadoom, Ryan R Brown, Christina Campbell, Andrew Jackson, Cliona McDowell, Mike Clarke, Fiona Copeland, Kathryn Ferguson, Rebecca H McLeese, Rory Convery, Adam T Hill, Anthony De Soyza, Mitra Shahidi, Martin Kelly, Mary Carroll, Timothy Gatheral, Muhammed Anwar, Jamie Duckers, William Flight, John R Hurst, A Alina Ionescu, Michael R Loebinger, Anita L Sullivan, James D Chalmers, Damian G Downey, Michael M Tunney, Brenda O'Neill, Daniel F McAuley, J Stuart Elborn, Judy M Bradley, Clifford C Taggart

Supplementary material

Supplementary methods

Sputum rheometry

Sputum aliquots that had previously been processed to remove contaminants were allowed to reach room temperature and were then homogenised at increasing speeds on a vortex until a torus was formed, at which point samples were vortexed for a further 30s to ensure homogeneity. This method of non-destructive vortexing has previously been shown to decrease within-sample variability without changing the mean viscoelastic values of the sputum (2). A positive displacement pipette was used to transfer 330µL to the lower geometry of a Rheonova Rheomuco® rotational plate rheometer (Rheonova, France) and the sample was then subjected to a strain sweep test at 37°C with oscillatory strain amplitudes ranging from 0.1% to 10,000% at 1Hz. Values for elastic modulus G', viscous modulus G" and crossover point σc were extracted and derived from the accompanying software (v1.4.1).

Sputum processing for ELISA

An aliquot of sputum was weighed and homogenised by repeated pipetting in a volume of 1X Dulbecco's PBS (14190144, Gibco, MD, USA) equal to 8 times the weight of the sample. Dispersed sputum was vortexed for 15s and left on a roller for 15min. Samples were centrifuged at 790xg for 10min at 4°C (without braking). Supernatants were transferred to a fresh tube and centrifuged at 1500xg for 10min at 4°C (without braking). Supernatants were then aliquoted and stored at -80°C until required for ELISA.

16S rRNA marker gene library preparation and MiSeq sequencing

16S rRNA marker-gene sequencing was carried out on the Illumina MiSeq platform following the manufacturer's standard workflow, with the exception that custom primers developed by the Earth Microbiome Project were used (3). Sequencing targeted the V4 region of the 16S rRNA gene to generate amplicon sequence variants (ASVs). In brief, amplicon PCR products were purified prior to sequencing using the AxyPrep Mag PCR Clean-up kit (Axygen, CA, USA). Thirty-five µL of resuspended magnetic beads were added to each PCR product in a 96-well plate, mixed thoroughly, and incubated at room temperature for 5 min. Following magnetic separation, the supernatant was removed, and beads were washed twice with 180µL 70% ethanol. After allowing the beads to air-dry, DNA was eluted in 50µL diethyl pyrocarbonate (DEPC)-treated water. The purified amplicons were quantified using the Quant-iT™ PicoGreen® dsDNA assay (Life Technologies, UK). Samples were then normalised to equimolar concentrations and pooled; for reactions that did not amplify, a constant input volume was included. The final library was quantified in triplicate with PicoGreen® prior to storage until sequencing on the Illumina MiSeq platform (Illumina, UK).

Bioinformatic processing of sequencing and statistical analysis

Paired-end 16S rRNA gene reads were processed with the DADA2 workflow (v1.22) (4) implemented in R (v4.1.2). Quality control involved trimming primer sequences (trimLeft=20), truncating reads at 250bp (forward) and 220bp (reverse), disallowing ambiguous bases (maxN=0), and applying expected error thresholds of 2 for both directions (maxEE=c(2,2). After dereplication (derepFastq()), error models were trained independently for forward and reverse reads (learnErrors()). Amplicon sequence variants (ASVs) were inferred with dada() and paired reads merged (mergePairs()) using a ≥12bp overlap with no mismatches permitted. Potential chimeras were identified and removed with removeBimeraDenovo() in consensus mode, resulting in retention of ~98% of input reads (sum(seqtab.nochim)/sum(seqtab)). Taxonomic assignment was performed against the

SILVA v138 reference database (assignTaxonomy()), with species-level annotations where available.

For downstream phylogenetic analysis, ASV sequences were aligned using DECIPHER (AlignSeqs()), converted to phyDat objects, and used to generate a maximum-likelihood tree with phangorn (optim.pml()). Taxonomic classification was performed using the SILVA v138 reference database, providing genus-level annotations. The ASV count matrix, taxonomy table, and phylogeny were integrated into a phyloseq object.

Contaminant identification and removal

To minimize the influence of potential contaminants, the *decontam* R package (v1.14.0) (5) was applied to the combined *phyloseq* object containing all study samples, including negative controls. Contaminant identification was carried out using the prevalence-based approach, which evaluates how frequently each ASV occurs in true biological samples compared with negative controls. Fisher's exact test was used to detect taxa disproportionately represented in the controls. Negative controls were annotated in the sample metadata under the variable "SampleType" = "NegativeControl".

Because DNA concentration measurements were unavailable, the frequency-based method within *decontam* could not be used at this stage. Nevertheless, the analytical structure was retained to allow subsequent re-analysis once DNA quantification data are obtained. The resulting list of potential contaminants was manually assessed through bar plot visualizations stratified by sample type to confirm consistent contamination trends.

Taxa recognized as common kit or reagent contaminants were excluded from the dataset, including *Bordetella*, *Hydrogenophilus*, *Acinetobacter*, *Kocuria*, *Spirosoma*, *Rubellimicrobium*, *Klebsiella*, *Tetragenococcus*, *Ralstonia*, *Psychrobacter*, *Shewanella*, *Chryseobacterium*, *Luteolibacter*, *Roseomonas*, *Alishewanella*, *Pelosinus*, *Bacillus*, *Methylobacterium-Methylorubrum*, *Sphingobacterium*, *Clostridium sensu stricto 9*, *Comamonas*, *Janthinobacterium*, *Enterococcus*, *Thermus*, *Dermacoccus*, *Sphingomonas*, *Frondihabitans*, *Lawsonella*, *Blastocatella*, *Cedecea*, *Rhizorhapis*, *Brevibacillus*, *Truepera*, *Citrobacter*, *Aeromonas*, *Brevundimonas*, *Thermoanaerobacter*, *Bacteroides*, *Gemmatimonas*, *Novosphingobium*, *Burkholderia*, *Polaromonas*, *Rhodococcus*, *Peptoclostridium*, *Acidiphilium*, *Afipia*, *Aridibacter*, *Pantoea*, *Anoxybacillus*, *Abditibacterium*, *RB41*, *Enhydrobacter*, *Dechlorosoma*, *Pajaroellobacter*, *Klenkia*, *Thermoanaerobacterium*, *Anaerospora*, *Paracoccus*, *Vibrio*, *Buchnera*, *Segetibacter*, *Neorhizobium*, *Rickettsiella*, *Caldicellulosiruptor*, *Candidatus Udaeobacter*, *Constrictibacter*, *Micrococcus*, *Bradyrhizobium*, *Nocardioides*, *1174-901-12*, and *Prevotellaceae UCG-001*.

After removing these taxa, ASVs were re-filtered within the *phyloseq* object, and relative abundances were recalculated using total-sum scaling (TSS) to account for differences in sequencing depth. This ensured that downstream analyses, such as diversity and ordination, were based on a decontaminated and compositionally accurate representation of the microbial communities.

Community analysis

Diversity analyses included measures of alpha diversity (richness [S], Shannon-Wiener diversity [H], Pielou's evenness, and dominance [relative]) and beta diversity (Bray-Curtis dissimilarity calculated on Hellinger-transformed data). Multivariate testing, including PERMANOVA, was carried out using the vegan (v2.5.7) (6), phyloseq (v1.38.0) (7) and microbiome (v1.19.1) (8) R packages.

To assess differences in microbial community structure between treatment groups over time, we evaluated four alpha-diversity metrics: richness, Shannon-Wiener diversity, Pielou's evenness, and dominance (relative). For each outcome, we used linear regression models including the baseline values of the metric as a covariate to adjust for inter-individual differences at baseline. This approach allowed estimation of adjusted mean values for each treatment group while accounting for baseline variation. Pairwise comparisons between treatment groups were performed using estimated marginal means (emmeans) with Tukey adjustment for multiple comparisons. Model residuals were examined to confirm assumptions of normality and homoscedasticity. Baseline values were consistently included as covariates and often emerged as the strongest predictors of follow-up measurements. All analyses were conducted in R (v4.1.2) using the lm() function for linear models and emmeans() from the emmeans package (v1.10.3) (9) for adjusted comparisons. Graphical presentation was done using the ggplot2 (v3.5.1) (10), MicrobiotaProcess (v.1.6.6) (11) and pheatmap (v1.0.12) (12) packages in R.

Supplementary Tables

Supplementary Table 1. Baseline characteristics at trial entry (all participants)

Baseline Characteristics		Treatment Group			
		HTS	Carbocisteine	HTS plus carbocisteine	Standard Care
		n=19	n=17	n=15	n=16
**Sex**	**Male**	13 (68.4%)	4 (23.5%)	8 (53.3%)	6 (37.5%)
	**Female**	6 (31.6%)	13 (76.5%)	7 (46.7%)	10 (62.5%)
**Age (years)**		59.7 (15.6)	70.0 (12.3)	69.1 (12.3)	66.7 (10.8)
**Ethnicity**	**White**	18 (94.7%)	17 (100.0%)	15 (100.0%)	15 (93.8%)
	**Asian/Asian British**	1 (5.3%)	0 (0.0%)	0 (0.0%)	0 (0.0%)
	**Chinese**	0 (0.0%)	0 (0.0%)	0 (0.0%)	1 (6.3%)
**Cigarette use**	**Never Smoked**	7 (36.8%)	11 (64.7%)	10 (66.7%)	7 (43.8%)
	**Ex-Smoker**				
	**Cigarettes**	11 (57.9%)	6 (35.3%)	5 (33.3%)	9 (56.3%)
	**Pipe**	1 (5.3%)	0 (0.0%)	0 (0.0%)	0 (0.0%)
**E-cigarette use**	**Never vaped**	19 (100.0%)	17 (100.0%)	15 (100.0%)	16 (100.0%)
**Weight (kg)**		81.1 (15.2)	72.1 (22.3)	78.7 (20.1)	80.4 (17.7)
**BSI**		5.3 (3.0)	8.3 (3.6) n=16	6.9 (4.1)	7.8 (3.9)
**FACED**		1.3 (1.3)	2.3 (1.8) n=16	1.9 (1.5) n=14	2.1 (1.6)
**HRQoL-B (Respiratory Domain)**		67.2 (14.5)	53.6 (20.0)	61.5 (16.5)	56.7 (16.7)

Mean (SD) presented for continuous variables and no. (%) for all categorical variables. If n is different to the total n per group (due to missing data) it is noted in the relevant cell. HTS: hypertonic saline; BSI: Bronchiectasis Severity Index; FACED: FEV1, age, chronic colonisation, extension, dyspnoea; HRQoL-B: Health-Related Quality of Life-Bronchiectasis score

Supplementary Table 2. Sputum viscoelasticity at 2 weeks after commencement of treatment (adjusted for baseline values)

	Treatment Group				p-value
	HTS n=12	Carbocisteine n=13	HTS plus carbocisteine n=8	Standard Care n=10	
Elastic modulus G' (Pa)	8.2 (4.3)	3.5 (4.2)	22.6 (5.4)	8.8 (4.7)	*0.002**
Viscous modulus G" (Pa)	2.0 (1.2)	1.0 (1.2)	5.9 (1.5)	1.7 (1.3)	*0.008**
Crossover point σc (Pa)	30.8 (7.1)	20.5 (7.2)	60.5 (9.0)	39.3 (7.8)	*<0.001**

Mean (SE) presented. p-value from ANCOVA. *Tukey HSD pairwise comparisons did not identify any significant differences. HTS: hypertonic saline.

Supplementary Table 3. Sputum viscoelasticity at 8 weeks after commencement of treatment (adjusted for baseline values)

	Treatment Group			
	HTS n=13	Carbocisteine n=15	HTS plus carbocisteine n=6	Standard Care n=8
Elastic modulus G' (Pa)	5.3 [-0.5, 16.9]	10.5 [2.8, 18.2]	13.0 [0.9, 25.1]	8.7 [-1.7, 19.1]
Viscous modulus G" (Pa)	1.2 [-0.6, 3.1]	2.6 [1.0, 4.3]	3.2 [0.5, 5.8]	1.8 [-0.5, 4.1]
Crossover point σc (Pa)	22.7 [-2.4, 47.8]	41.0 [16.9, 65.2]	42.5 [5.0, 80.0]	38.1 [6.4, 69.8]

Mean [95% CI] presented. HTS: hypertonic saline.

Supplementary Table 4. Sputum biomarkers at 2 weeks after commencement of treatment (adjusted for baseline values)

	Treatment Group			
	HTS n=6	Carbocisteine n=10	HTS plus carbocisteine n=3	Standard Care n=2
IL-6 (pg/mL)	1.7 [0.1, 3.3]	2.8 [1.6, 4.1]	1.3 [-0.3, 4.2]	1.7 [-1.0, 4.5]
IL-8 (pg/mL)	32517.9 [21139.7, 43896.1]	18425.5 [9526.5, 27324.4]	30803.0 [15889.9, 45716.1]	22403.1 [4518.1, 40288.1]
8-isoprostane (pg/mL)	51.6 [30.6, 72.6]	47.6 [31.1, 64.0]	61.7 [32.7, 90.8]	46.8 [12.1, 81.5]

Mean [95% CI] presented. HTS: hypertonic saline.

Supplementary Table 5. Sputum biomarkers at 8 weeks after commencement of treatment (adjusted for baseline values)

	Treatment Group			
	HTS n=7	Carbocisteine n=10	HTS plus carbocisteine n=2	Standard Care n=2
IL-6 (pg/mL)	1.2 [0.1, 2.4]	1.8 [0.8, 2.8]	1.1 [-1.1, 3.2]	1.3 [-0.9, 3.4]
IL-8 (pg/mL)	33028.9 [10688.9, 55368.9]	33911.4 [15032.5, 52790.2]	23654.8 [-15292.3, 62601.8]	56875.9 [18272.6, 95479.1]
8-isoprostane (pg/mL)	56.2 [16.7, 95.8]	76.9 [43.7, 110.2]	40.4 [-35.2, 116.0]	68.5 [-5.4, 142.4]

Mean [95% CI] presented. HTS: hypertonic saline.

Supplementary Table 6. Sputum bacterial community composition at 2 weeks after commencement of treatment (adjusted for baseline values)

	Treatment Group			
	**HTS n=10**	**Carbocisteine n=13**	**HTS plus carbocisteine n=10**	**Standard Care n=6**
Shannon–Wiener diversity	1.56 [1.23, 1.89]	1.07 [0.77, 1.36]	1.13 [0.81, 1.45]	1.39 [0.94, 1.85]
Pielou's evenness	0.40 [0.32, 0.48]	0.28 [0.21, 0.35]	0.29 [0.21, 0.37]	0.34 [0.23, 0.46]
Richness	46.32 [41.55, 51.08]	44.33 [40.02, 48.65]	45.37 [40.70, 50.04]	47.23 [40.63, 53.83]
Relative dominance	0.51 [0.38, 0.63]	0.69 [0.58, 0.80]	0.73 [0.61, 0.85]	0.61 [0.44, 0.78]

Mean [95% CI] presented. HTS: hypertonic saline.

Supplementary Table 7. Sputum bacterial community composition at 8 weeks after commencement of treatment (adjusted for baseline values)

	Treatment Group			
	**HTS n=10**	**Carbocisteine n=15**	**HTS plus carbocisteine n=5**	**Standard Care n=8**
**Shannon–Wiener diversity**	1.59 [1.23, 1.96]	1.18 [0.89, 1.48]	1.16 [0.67, 1.65]	1.41 [0.95, 1.87]
**Pielou's evenness**	0.41 [0.32, 0.50]	0.30 [0.23, 0.37]	0.29 [0.17, 0.41]	0.38 [0.27, 0.49]
**Richness**	46.15 [40.15, 52.14]	45.68 [40.78, 50.58]	51.71 [43.47, 59.94]	37.10 [29.17, 45.03]
**Relative dominance**	0.53 [0.41, 0.64]	0.66 [0.57, 0.76]	0.65 [0.49, 0.81]	0.60 [0.45, 0.75]

Mean [95% CI] presented. HTS: hypertonic saline.

Supplementary Table 8. Post-hoc groupwise analysis of primary outcome (rheometry at 2 weeks) adjusted for baseline values

	Comparison				Comparison			
	**HTS (n=20)**	**No HTS (n=23)**	**Mean difference (95% CI)**	**p-value**	**Carbocisteine (n=21)**	**No carbocisteine (n=22)**	**Mean difference (95% CI)**	**p-value**
Elastic modulus G' (Pa)	13.8 (3.5)	5.9 (3.2)	7.9 (-1.7, 17.5)	0.105	10.8 (3.5)	8.5 (3.4)	2.3 (-7.5, 12.2)	0.637
Viscous modulus G" (Pa)	3.5 (1.0)	1.3 (0.9)	2.2 (-0.5, 4.8)	0.107	2.8 (1.0)	1.8 (0.9)	1.0 (-1.7, 3.7)	0.471
Crossover point σc (Pa)	41.6 (6.1)	29.7 (5.7)	11.9 (-5.3, 29.1)	0.170	36.4 (6.0)	34.0 (5.8)	2.4 (-14.6, 19.3)	0.778

Mean (SE) presented. p-value from ANCOVA. HTS: hypertonic saline.

Supplementary Table 9. Post-hoc groupwise analysis of secondary outcomes adjusted for baseline values

	Comparison				Comparison			
	HTS	No HTS	Mean difference (95% CI)	p-value	Carbocisteine	No carbocisteine	Mean difference (95% CI)	p-value
**Rheometry**								
Elastic modulus G' (Pa) 8 weeks	7.7 (3.3) n=19	9.9 (3.0) n=23	-2.2 (-11.3, 7.0)	0.638	11.2 (3.11) n=21	6.6 (3.11) n=21	4.6 (-4.3, 13.5)	0.303
Viscous modulus G" (Pa) 8 weeks	1.9 (0.7) n=19	2.3 (0.7) n=23	-0.5 (-2.5, 1.6)	0.628	2.8 (0.7) n=21	1.4 (0.7) n=21	1.3 (-0.6, 3.3)	0.170
Crossover point σc (Pa) 8 weeks	28.8 (10.3) n=19	40.2 (9.3) n=23	-11.4 (-40.0, 17.3)	0.428	41.4 (9.6) n=21	28.6 (9.6) n=21	12.8 (-14.7, 40.4)	0.352
**Biomarkers**								
IL-6 (pg/mL) 2 weeks	1.8 (0.6) n=9	2.6 (0.5) n=12	-0.8 (-2.5, 0.8)	0.313	2.6 (0.5) n=13	1.7 (0.6) n=8	0.9 (-0.8, 2.6)	0.288
IL-6 (pg/mL) 8 weeks	1.2 (0.5) n=9	1.7 (0.4) n=12	-0.5 (-1.8, 0.8)	0.423	1.7 (0.4) n=12	1.2 (0.5) n=9	0.4 (-0.9, 1.7)	0.512
IL-8 (pg/mL) 2 weeks	31724.5 (4193.5) n=9	19254.7 (3544.0) n=12	12469.8 (9.7, 24930.0)	0.050	22059.8 (3539.5) n=13	28725.0 (4625.2) n=8	-6665.3 (-19484.4, 6153.9)	0.289
IL-8 (pg/mL) 8 weeks	29840.6 (9174.0) n=9	38567.6 (7789.5) n=12	-8727.0 (-35664.9, 18210.8)	0.505	31448.2 (7702.0) n=12	39333.2 (9037.0) n=9	-7885.0 (-34161.5, 18392.0)	0.536
8-isoprostane (pg/mL) 2 weeks	55.0 (7.9) n=9	47.5 (6.7) n=12	7.5 (-15.4, 30.5)	0.499	51.3 (6.3) n=13	49.7 (8.1) n=8	1.6 (-20.5, 23.6)	0.884
8-isoprostane (pg/mL) 8 weeks	52.9 (15.7) n=9	75.4 (13.6) n=12	-22.5 (-66.6, 21.6)	0.298	70.8 (13.8) n=12	59.1 (15.9) n=9	11.7 (-32.6, 56.0)	0.586
**Bacterial community composition**								
Shannon-Wiener diversity 2 weeks	1.6 (0.2) n=21	1.4 (0.2) n=20	0.2 (-0.4, 0.7)	0.553	1.1 (0.1) n=19	1.4 (0.2) n=22	-0.3 (-0.8, 0.2)	0.233
Shannon-Wiener diversity 8 weeks	1.6 (0.2) n=18	1.4 (0.2) n=18	0.2 (-0.4, 0.7)	0.521	1.2 (0.1) n=17	1.4 (0.2) n=19	-0.2 (-0.8, 0.3)	0.414
Pielou's evenness 2 weeks	0.4 (0) n=21	0.3 (0.1) n=20	0.1 (-0.1, 0.2)	0.429	0.3 (0) n=19	0.3 (0.1) n=22	-0.1 (-0.2, 0.1)	0.306
Pielou's evenness 8 weeks	0.4 (0) n=18	0.4 (0.1) n=18	0.0 (-0.1, 0.2)	0.699	0.3 (0) n=17	0.4 (0.1) n=19	-0.1 (-0.2, 0.1)	0.237
Richness	46.3 (2.3)	47.2 (3.2)	-0.9 (-8.7,	0.819	44.3 (2.1)	47.2 (3.2)	-2.9 (-10.5,	0.463

2 weeks	n=21	n=20	6.9)		n=19	n=22	4.7)	
Richness 8 weeks	46.1 (2.9) n=18	37.1 (3.9) n=18	9 (-0.1, 18.2)	0.062	45.7 (2.4) n=17	37.1 (3.9) n=19	8.6 (-0.8, 18)	0.084
Relative dominance 2 weeks	0.5 (0.1) n=21	0.6 (0.1) n=20	-0.1 (-0.3, 0.1)	0.306	0.7 (0.1) n=19	0.6 (0.1) n=22	0.1 (-0.1, 0.3)	0.428
Relative dominance 8 weeks	0.5 (0.1) n=18	0.6 (0.1) n=18	-0.1 (-0.3, 0.1)	0.415	0.7 (0) n=17	0.6 (0.1) n=19	0.1 (-0.1, 0.2)	0.502

Mean (SE) presented. p-value from ANCOVA. n number for each condition presented in corresponding cell. HTS: hypertonic saline.

Supplementary Figures

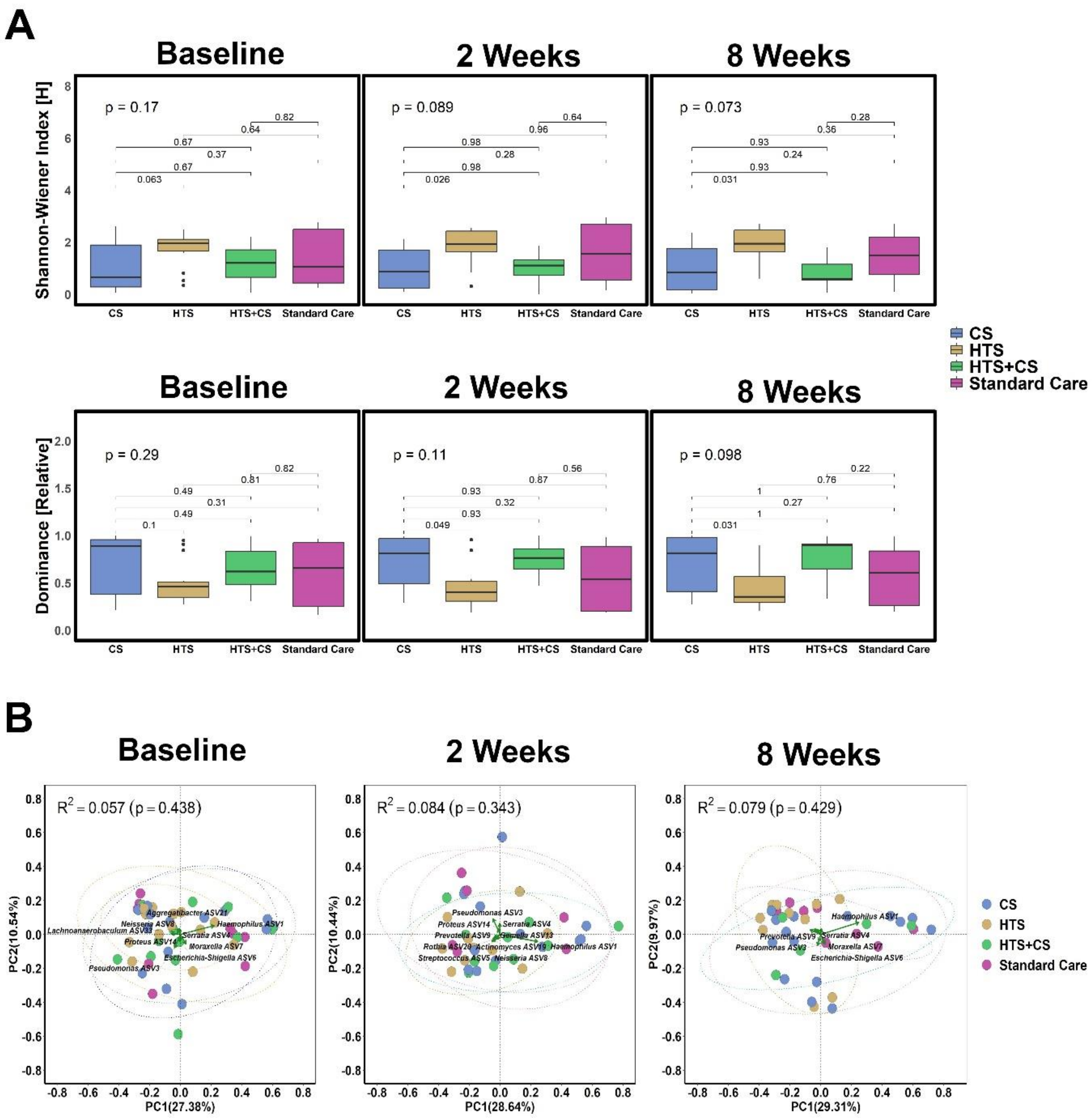

Supplementary Figure 1. (A) Shannon-Wiener Index and relative dominance across treatment groups over time. The upper panel displays the Shannon-Wiener Index [H], a measure of alpha diversity, at baseline, 2 weeks, and 8 weeks post-treatment initiation. The lower panel shows the Dominance [Relative] index, indicating the relative abundance of the most dominant taxa, at the same time points. Data are presented as box-whiskers plots showing mean ± 95% CI. (B) PCoA plots illustrating the clustering of microbiome samples based on taxa composition at baseline, 2 weeks, and 8 weeks. Each point represents an individual sample, coloured according to its treatment group: CS (carbocisteine), HTS (hypertonic saline), HTS+CS (hypertonic saline plus carbocisteine), and standard care alone. The ellipses represent 95% confidence intervals around the group centroids. The R^2 value indicates the proportion of variance explained by the displayed principal components, and the number in parentheses represents the corresponding p-values. P-value <0.05 denote statistical significance.

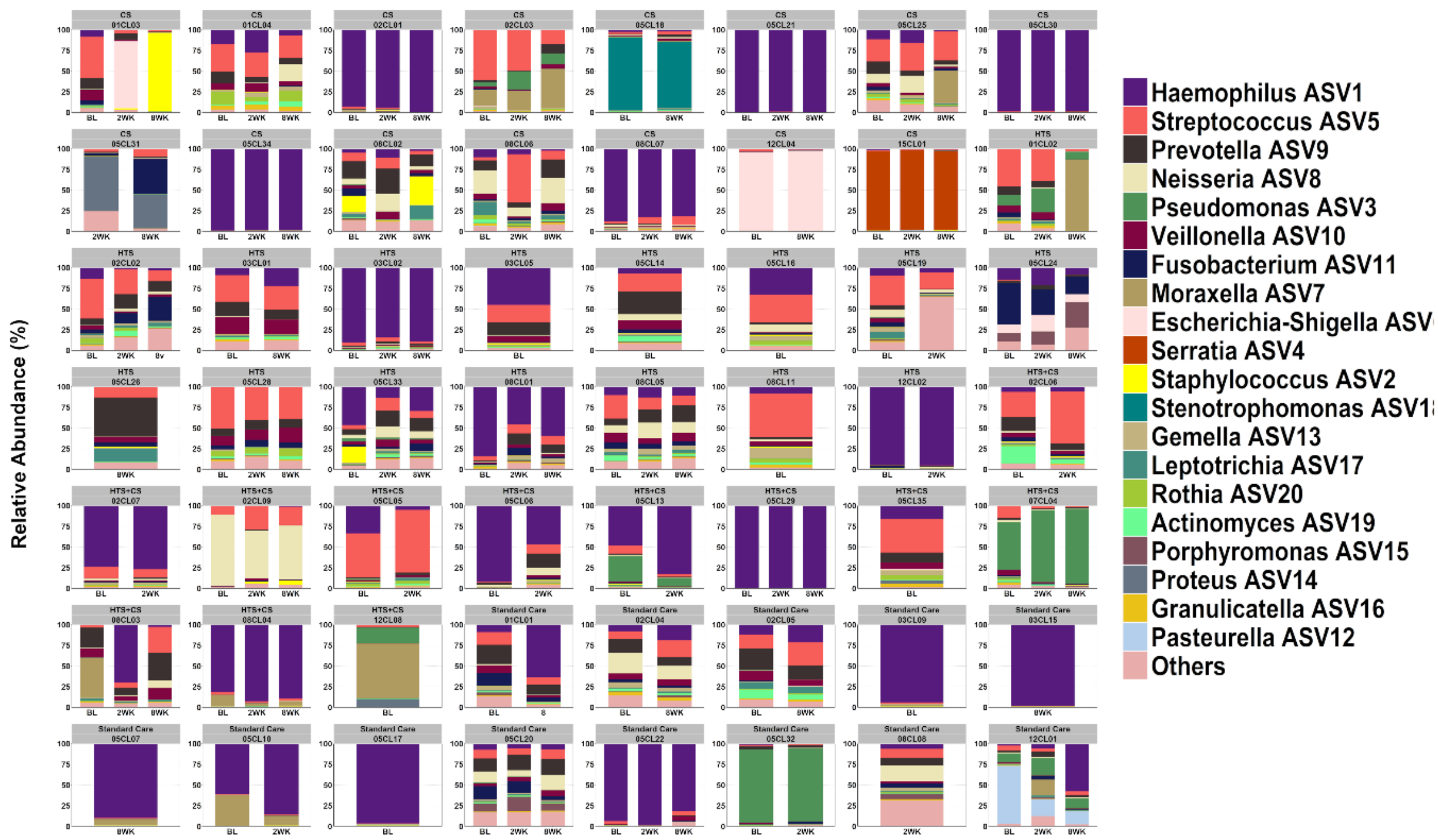

Supplementary Figure 2. Bacterial taxa relative abundance by treatment group and sample time point. The relative abundance of bacterial taxa was calculated and presented as stacked bar plots. Each plot represents an individual patient, and each bar represents a time point. The treatment received by that patient is noted on the plot. Data for all available samples are presented; due to limited sample volume, not all patients had microbiome data available for all three time points. BL: baseline; 2WK: 2 weeks after randomisation; 8WK: 8 weeks after randomisation; HTS: hypertonic saline; CS: carbocisteine; HTS+CS: hypertonic saline plus carbocisteine.

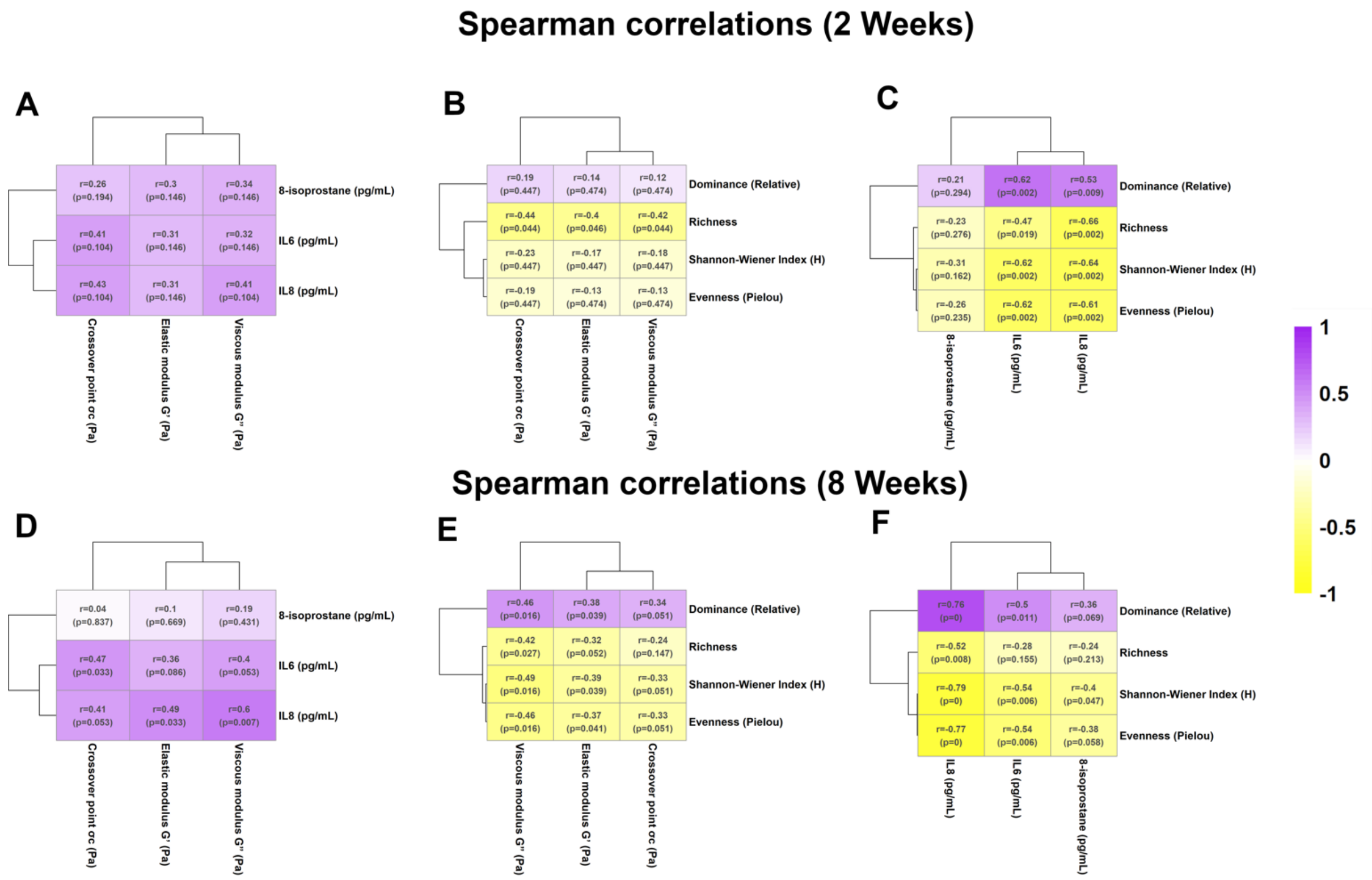

Supplementary Figure 3. Correlations in CLEAR-EME sputum at 2 weeks and 8 weeks after commencement of treatment. Sputum properties at (A-C) 2 weeks and (D-F) 8 weeks after the commencement of treatment were correlated. Sputum viscoelasticity (elastic modulus G', viscous modulus G" and crossover point σc) and markers of inflammation (IL-6 and IL-8) and oxidative stress (8-isoprostane) were correlated with alpha-diversity indices of bacterial community composition (Shannon-Wiener diversity, Pielou's evenness, richness and relative dominance). Each cell contains the Spearman correlation coefficient (r) and the corresponding p-value. The colour intensity represents the magnitude and direction of the correlation, with purple indicating positive correlations and yellow indicating negative correlations. Correlations were considered statistically significant if $p<0.05$.

References

1. King M, Dasgupta B, Tomkiewicz RP, Brown NE. Rheology of cystic fibrosis sputum after in vitro treatment with hypertonic saline alone and in combination with recombinant human deoxyribonuclease I. Am J Respir Crit Care Med. 1997;156(1):173–7.

2. Esteban Enjuto L, Robert de Saint Vincent M, Maurin M, Degano B, Bodiguel H. Sputum handling for rheology. Sci Rep [Internet]. 2023 Dec 1 [cited 2025 Oct 8];13(1):1–10. Available from: https://www.nature.com/articles/s41598-023-34043-9

3. Greg Caporaso J, Ackermann G, Apprill A, Bauer M, Berg-Lyons D, Betley J, et al. EMP 16S Illumina Amplicon Protocol v1 [Internet]. 2018 [cited 2025 Oct 27]. Available from: https://www.protocols.io/view/emp-16s-illumina-amplicon-protocol-nuudeww

4. Callahan BJ, McMurdie PJ, Rosen MJ, Han AW, Johnson AJA, Holmes SP. DADA2: High-resolution sample inference from Illumina amplicon data. Nat Methods [Internet]. 2016 Jun 29 [cited 2025 Oct 23];13(7):581–3. Available from: https://www.nature.com/articles/nmeth.3869

5. Davis NM, Proctor DM, Holmes SP, Relman DA, Callahan BJ. Simple statistical identification and removal of contaminant sequences in marker-gene and metagenomics data. bioRxiv [Internet]. 2018 Jul 25 [cited 2025 Oct 23];221499. Available from: https://www.biorxiv.org/content/10.1101/221499v2

6. Oksanen J, Blanchet FG, Friendly M, Kindt R, Legendre P, Michin PR, et al. vegan: Community Ecology Package. R package version 2.5-7 [Internet]. 2020 [cited 2025 Oct 23]. Available from: https://CRAN.R-project.org/package=vegan

7. McMurdie PJ, Holmes S. phyloseq: An R Package for Reproducible Interactive Analysis and Graphics of Microbiome Census Data. PLoS One [Internet]. 2013 Apr 22 [cited 2025 Oct 23];8(4):e61217. Available from: https://journals.plos.org/plosone/article?id=10.1371/journal.pone.0061217

8. Lahti L, Shetty S, et al. Tools for microbiome analysis in R. Microbiome package version 1.31.2 [Internet]. Bioconductor; 2017 [cited 2025 Oct 23]. Available from: http://microbiome.github.com/microbiome

9. Lenth R V. emmeans: Estimated Marginal Means, aka Least-Squares Means. R package version 1.10.3 [Internet]. 2024 [cited 2025 Oct 23]. Available from: https://CRAN.R-project.org/package=emmeans

10. Wickham H. ggplot2: Elegant Graphics for Data Analysis. New York: Springer-Verlag; 2016.

11. Xu S, Zhan L, Tang W, Wang Q, Dai Z, Zhou L, et al. MicrobiotaProcess: A comprehensive R package for deep mining microbiome. The Innovation [Internet]. 2023 Mar 13 [cited 2025 Oct 23];4(2):100388. Available from: https://www.sciencedirect.com/science/article/pii/S2666675823000164

12. Kolde R. pheatmap: Pretty Heatmaps. R package version 1.0.12 [Internet]. 2019 [cited 2025 Oct 23]. Available from: https://CRAN.T-project.org/package=pheatmap
